# Assessment of Favipiravir and Remdesivir in Combination for SARS-CoV-2 Infection in Syrian Golden Hamsters

**DOI:** 10.3390/v16121838

**Published:** 2024-11-27

**Authors:** Megan Neary, Eduardo Gallardo-Toledo, Joanne Sharp, Joanne Herriott, Edyta Kijak, Chloe Bramwell, Helen Cox, Lee Tatham, Helen Box, Paul Curley, Usman Arshad, Rajith K. R. Rajoli, Henry Pertinez, Anthony Valentijn, Shaun H. Pennington, Claire H. Caygill, Rose C. Lopeman, Giancarlo A. Biagini, Anja Kipar, James P. Stewart, Andrew Owen

**Affiliations:** 1 Department of Pharmacology and Therapeutics, Institute of Systems, Molecular and Integrative Biology, University of Liverpool, Liverpool L69 3BX, UK; mneary098@gmail.com (M.N.); e.gallardo@liverpool.ac.uk (E.G.-T.); momeejl2@liverpool.ac.uk (J.S.); flanders@liverpool.ac.uk (J.H.); e.kijak@liverpool.ac.uk (E.K.); c.bramwell@liverpool.ac.uk (C.B.); coxy67@liverpool.ac.uk (H.C.); l.tatham@liverpool.ac.uk (L.T.); helen-box@hotmail.co.uk (H.B.); pcurley@liverpool.ac.uk (P.C.); u.arshad2@liverpool.ac.uk (U.A.); rkrajoli@liverpool.ac.uk (R.K.R.R.); pertinez@liverpool.ac.uk (H.P.); ajv@liverpool.ac.uk (A.V.); 2 Centre of Excellence in Long-Acting Therapeutics (CELT), University of Liverpool, Liverpool L69 3BX, UK; 3 Centre for Drugs and Diagnostics, Department of Tropical Disease Biology, Liverpool School of Tropical Medicine, Liverpool L3 5QA, UK; shaun.pennington@lstmed.ac.uk (S.H.P.); claire.caygill@lstmed.ac.uk (C.H.C.); rose.lopeman@lstmed.ac.uk (R.C.L.); giancarlo.biagini@lstmed.ac.uk (G.A.B.); 4 Department of Infection Biology & Microbiomes, Institute of Infection, Veterinary and Ecological Sciences, University of Liverpool, Liverpool L69 3BX, UK; anja.kipar@uzh.ch (A.K.); jpstewar@liverpool.ac.uk (J.P.S.); 5 Laboratory for Animal Model Pathology, Institute of Veterinary Pathology, Vetsuisse Faculty, University of Zurich, 8057 Zurich, Switzerland

**Keywords:** SARS-CoV-2, favipiravir, remdesivir, combination therapy

## Abstract

Favipiravir (FVP) and remdesivir (RDV) have demonstrable antiviral activity against SARS-CoV-2. Here, the efficacy of FVP, RDV, and FVP with RDV (FVP + RDV) in combination was assessed in Syrian golden hamsters challenged with SARS-CoV- 2 (B.1.1.7) following intraperitoneal administration. At day 4 post infection, viral RNA and viral antigen expression were significantly lower in lungs for all three treatment groups compared to the sham treatment. Similarly, viral titres in the lungs were lower in all treatment groups compared to the sham treatment. The FVP + RDV combination was the only treatment group where viral RNA in nasal turbinate and lung, virus titres in lung, and viral antigen expression (lung) were all lower than those for the sham treatment group. Moreover, lower viral titre values were observed in the FVP + RDV group compared to other treatment groups, albeit only significantly lower in comparison to those in the RDV-only-treated group. Further assessment of the potential utility of FVP in combination with RDV may be warranted. Future studies should also consider whether the combination of these two drugs may reduce the speed at which drug resistance mutations are selected.

## 1. Introduction

Since the first report of human infection by SARS-CoV-2 in December 2019, more than 7.1 million global deaths from COVID-19 have been reported. During this period, new outbreaks have occurred due to the appearance of new variants of concern (VOCs), which are characterised by showing modified features of the original virus, such as greater replication rates and altered immunogenicity [1]. Vaccine roll-out has been highly successful in controlling the pandemic [2], but continued development of treatment options remains highly warranted, particularly since highly effective monoclonal antibodies have been compromised for recently emerged variants [3].

Favipiravir (FVP) was originally developed as an antiviral for influenza [4], and remdesivir (RDV) was originally developed for hepatitis C virus, but both exhibit a broad-spectrum of activity against many viruses [5]. Both antivirals are nucleotide analogues that are incorporated into the replicated RNA by the viral RNA-dependent RNA polymerase. While FVP induces lethal mutagenesis [4], RDV causes delayed chain termination to exert its antiviral efficacy [5]. Several clinical trials have reported efficacy of FVP and RDV as monotherapies for treatment of SARS-CoV-2 infection, demonstrating a reduction in recovery times and a notable clinical improvement in mild-to-moderate disease [6,7]. However, robust evidence of efficacy from randomised controlled trials is only available for RDV, and as such RDV but not FVP is recommended by WHO [8]. The combination of antiviral medications has been proposed for the prevention and treatment of COVID-19 [9,10]. The combination of drugs has been previously used against other viruses such as hepatitis C virus (HCV) and human immunodeficiency virus (HIV), demonstrating greater efficacy compared to monotherapies and mitigating the resistance risk [11]. In the case of COVID-19, different in vivo studies have demonstrated or ruled out greater efficacy when combining two or more antivirals, an antiviral with a pharmacoenhancer, or antiviral drugs with disease-modifying agents [12,13,14,15].

Syrian golden hamsters (*Mesocricetus auratus*) are a well established in vivo model for the study of SARS-CoV-2 transmission [16,17]. Hamsters are susceptible to viral respiratory infection and show clinical/pathological features similar to those seen in human patients [16]. This model allows the evaluation of virological efficacy of putative antiviral drugs under different experimental conditions [12,13,15]. Furthermore, it has been used to determine and compare the efficacy of monotherapies and drug combinations [13,14,18].

This study sought to investigate the efficacy of FVP and RDV alone and in combination following intraperitoneal administration to Syrian golden hamsters. The presented data demonstrate that all treatments reduced infection and replication in the lungs of infected animals. Histological analysis revealed no effect of treatments on the viral host cell pattern.

## 2. Materials and Methods

### 2.1. Materials and Animals

FVP and RDV were purchased from BIOSYNTH LTD (Newbury, UK). Sucrose and hydroxypropyl methylcellulose (HPMC; Mn = 10,000 g/mol) were purchased from Merck Life Science (Gillingham, UK). Male Syrian golden hamsters (*Mesocricetus auratus*) were obtained from Janvier Labs (Essex, UK). Transmission cages were purchased from Tecniplast UK Ltd. (Leicester, UK). Precellys CKmix lysing tubes and bead mill homogeniser were purchased from Bertin Technologies (Montigny-le-Bretonneux, France) and Fisher Scientific (Loughborough, UK), respectively. Nanodrop, TRIzol reagent, TURBO DNA-free^TM^ kits, GlycoBlue^TM^, Phasemaker^TM^ tubes, and Pierce™ BCA Protein Assay Kit were obtained from ThermoFisher Scientific (Runcorn, UK). While GoTaq^®^ Probe 1-Step RT-qPCR System was purchased from Promega (Fitchburg, WI, USA), both CDC RUO 2019-nCoV_N_Positive Control and SARS-CoV-2 (2019-nCoV) CDC qPCR Probe Assay were obtained from IDT (New-ark, NJ, USA). A Chromo4^TM^ Real-Time PCR Detector was purchased from Bio-Rad (Kidlington, UK). High-glucose Dulbecco’s modified Eagle’s medium (DMEM), Eagle’s Minimum Essential Medium (EMEM), heat-inactivated foetal bovine serum (HI FBS), and Dulbecco’s Phosphate-buffered saline (PBS) were purchased from Gibco^TM^, ThermoFisher Scientific (Runcorn, UK). In addition, foetal bovine serum (FBS), 1% penicillin/streptomycin, 2.3% crystal violet powder and solution, and 10% neutral buffered formalin solution were obtained from Merck Life Science (Gillingham, UK). Further, 2% UltraPure^TM^ Low Melting Point Agarose was purchased from Invitrogen, ThermoFisher Scientific (Runcorn, UK). For immunohistology, the rabbit anti-SARS-CoV nucleoprotein antibody was purchased from Rocklands (Pottstown, PA, USA); the peroxidase blocking buffer, the Envision+System HRP Rabbit, and the diaminobenzidine were obtained from Agilent DAKO (Carpinteria, CA, USA).

### 2.2. Virus Isolates

Human nCoV19 isolate/England/202012/01B (lineage B.1.1.7 Alpha variant) was obtained from the National Infection Service at Public Health England, Porton Down, UK, via the European Virus Archive (catalogue code 004V-04032). This was supported by the European Virus Archive GLOBAL (EVA-GLOBAL) project that has received funding from the European Union’s Horizon 2020 research and innovation programme under grant agreement No 871029. SARS-CoV-2 B.1.1.7 Alpha variant exhibits comparable fitness to other VOC in hamsters [17].

### 2.3. Ethical and Risk Assessments Approval

Animal study and procedures were carried out approved by the local University of Liverpool Animal Welfare and Ethical Review Board in accordance with UK Home Office Animals Scientific Procedures Act (ASPA, 1986) under the UK Home Office Project License PP4715265. Additionally, all work involving SARS-CoV-2 was performed within a containment level 3 (CL3) facility. All standard operating procedures (SOPs) and risk assessments were approved by the UK Health and Safety Executive and the University of Liverpool Biohazards Sub-Committee before the beginning of the study.

### 2.4. Assessment of the Efficacy of FVP and RDV Alone and in Combination Against SARS-CoV-2 in Syrian Golden Hamsters

Male Syrian golden hamsters (80–100 g) were randomly assigned to groups of four (random block study design). Animals were housed in ventilated cages with environmental enrichment, free access to food and water, and a 12 h light/dark cycle at 21 °C ± 2 °C. Prior to study, hamsters were acclimatised for 7 days prior to study initiation under SPF barrier conditions. All animals were weighed and monitored daily for any other clinical signs in addition to weight loss throughout the experiment. The average weight at each time point for every group was represented as a percentage in relation to the average weight on day −1 before infection within that specific group.

To assess the efficacy of FVP and RDV alone and in combination against SARS-CoV-2 infection, 4 hamsters per group were administered at day −1 as follows: intramuscular injection of sham treatment (150 µL of sucrose and HPMC/thigh, 300 µL total, sham treatment #1-4), or intraperitoneal injection of 150 mg/kg of FVP (twice daily, 300 mg/kg/day, FVP #1-4), 15 mg/kg RDV (once daily, 15 mg/kg/day, RDV #1-4), or 150 mg/kg FVP with 15 mg/kg of RDV (twice/once daily, respectively, FVP + RDV #1-4). All hamsters in each group (except the sham treatment group) were dosed according to their respective dosing regimen from day −1 to day 3 (Figure S1). At day 0, treatment groups were dosed, and then all groups were intranasally inoculated with 100 µL of 1 × 10^2^ PFU of SARS-CoV-2 B.1.1.7 Alpha in PBS under anaesthesia (3% isoflurane). All hamsters were culled at 4 days post infection (dpi) via a lethal intraperitoneal injection of pentobarbitone, and immediate exsanguination from the heart through cardiac puncture. Nasal turbinate samples were taken for PCR analysis. Samples from the right lung were used for downstream PCR and plaque assay, whereas the left lung was dissected and fixed for histological analysis (10% buffered formalin).

### 2.5. Quantification of SARS-CoV-2 N-RNA via qPCR

Quantification of total SARS-CoV-2 viral RNA (N-gene) was performed by quantitative (q)RT-PCR following an adaptation from the CDC 2019-Novel Coronavirus (2019-nCoV) Real-Time PCR Diagnostic Panel [19]. Sample inactivation, RNA extraction, and RNA quantification were carried out as previously described [12,13]. Briefly, samples from the nasal turbinate and right lung were inactivated and homogenised in TRIzol reagent. Total RNA was extracted using Phasemaker^TM^ tubes according to the manufacturer’s instructions. Subsequently, the recovered RNA was quantified using a Nanodrop, and DNAse treated using the TURBO DNA-free^TM^ kit according to the manufacturer’s instructions.

The qPCR standards were prepared from 10-fold serial dilutions of the CDC RUO 2019-nCoV_N_Positive Control (IDT) and 18S positive control [20]. Separately, DNAse-treated RNA, nuclease-free water (negative control), and the qPCR standards were mixed with the reaction master mix (GoTaq^®^ Probe + 18S RNA primers and probe sequences + N1 primer/probe mix from the SARS-CoV-2 (2019-nCoV) CDC qPCR Probe Assay [21]), and run using a Chromo4^TM^ Real-Time PCR Detector (Bio-Rad). The thermal cycling conditions for the qRT-PCR reactions were as follows: 1 cycle of 45 °C for 15 min, 1 cycle of 95 °C for 2 min, and 45 cycles of 95 °C for 3 s (Step 1) and 55 °C for 30 s (Step 2).

Finally, N1-RNA data were normalised to 18S data for subsequent quantitation. The limit of detection (LOD) for the assay was defined as a N1-RNA value of ≤2 copies/reaction and a PCR Ct value cut-off of ≥32 cycles. This cut-off was selected based on previously published data, which demonstrated that PCR Ct values between 17 and 32 represent culturable virus amounts [22,23].

### 2.6. Plaque Assay

Sample homogenization procedures, as well as Vero E6 cell maintenance protocols, were the same as used previously [12]. Of note, the sham treatment group samples were processed separately from the treatment groups (FPV, RDV, FPV + RDV) on different days, meaning that statistical comparisons between the sham treatment group and the treatment groups could not be conducted.

For the sham treatment group, homogenized samples were thawed, diluted in EMEM (1:4, 1:20, 1:100, 1:500, 1:2500, and 1:125,000) and layered over confluent Vero E6 cells in 100 μL volumes, in triplicate, in 96-well plates. A quantity of 100 μL semi-solid medium (EMEM supplemented with 4% HI FBS and 0.1% agarose) was then added to each well. Protocols for plate incubation (72 h), fixation (4% paraformaldehyde), and staining (70% *v*/*v* H_2_O, 10% *v*/*v* ethanol, 20% *v*/*v* methanol, and 0.25% crystal violet powder) were the same as previously described [24].

For the FVP-, RDV-, and FVP + RDV-treated groups, homogenised tissue samples were serial diluted (1–10^−3^ of the virus titre) in maintenance media (DMEM high glucose, 2% FBS), and layered over Vero E6 cells, in 24-well plates (100 µL, in duplicate). After one hour of incubation (37 °C, 5% CO_2_), 0.5 mL of freshly prepared overlay (4:1 of maintenance media:2% UltraPure^TM^ Low Melting Point Agarose) was added to each well. Plates were incubated (72 h), fixed (10% neutral buffered formalin), and stained (2.3% crystal violet solution) as previously described [12].

The number of formed plaques per well were counted manually at the highest countable concentration. The following formula was applied to determine the average PFU per mL (PFU/mL):

(#Plaques)/(d × V) = PFU/mL

d = Dilution factor

V = Volume of diluted virus added to the well (mL).

Finally, the results obtained in PFU/mL were normalized to PFU/µg of protein. For this, aliquots from the homogenized samples used for plaque assays (50 µL) were inactivated with 450 µL of PBS containing 0.5% Triton-X. Once inactivated, the total protein per sample (µg of protein/mL) was determined using the Pierce™ BCA Protein Assay Kit according to the manufacturer’s instructions.

### 2.7. Histological, Immunohistological, and Morphometric Analyses

After fixation in 10% buffered formalin for 48 h, the left lungs were stored in 70% ethanol until further processing. Two longitudinal sections were prepared and paraffin wax was routinely embedded. Consecutive sections (3–5 µm) were prepared and stained with haematoxylin eosin (HE) for histological examination or subjected to immunohistological staining to detect SARS-CoV-2 antigen (performed in an autostainer; Agilent DAKO), using the horseradish peroxidase (HRP) method and rabbit anti-SARS-CoV nucleocapsid protein (Rockland) as previously described [25,26].

For quantification of viral nucleocapsid protein (NP) expression in the lung, a morphometric analysis was undertaken on the slides stained for SARS-CoV-2 NP, as previously described [26]. The stained slides were scanned (NanoZoomer 2.0-HT; Hamamatsu, Hamamatsu City, Japan) and the lung sections of each animal quantitatively analysed using the Visiopharm 2022.01.3.12053 software (Visiopharm, Hoersholm, Denmark). The morphometric analysis served to quantify the area, in all lung sections of an animal, that showed immunostaining for SARS-CoV-2 NP. In Visiopharm, for each section, the lung was manually outlined and annotated as a Region of Interest (ROI), manually excluding artefactually altered areas. The manual tissue selection was further refined with an Automated Analysis Protocol (APP) based on a Decision Forest classifier, with the pixels from the Regions of Interest (ROIs) being ultimately classified as either “Tissue” or “Background”. This new “Tissue” ROI, regrouping the different lung samples analysed for each animal, was further quantified by executing two APPs successively. The first APP was based on a Threshold classifier and served to detect and outline areas with immunostaining. The second APP then measured both the surface of the immunostained area (µm^2^) and the surface of the “Tissue” ROI (µm^2^). The percentage of immunostained area (%), expressed as the ratio between the immunostained area and the total area, was obtained for each animal in Excel (Microsoft Office 2019; Microsoft, Redmond, Washington, United States), according to the following formula: ([positive area (µm^2^)]/[total area (µm^2^)]) × 100.

### 2.8. Statistical Analysis

A power calculation (NC3Rs Experimental Design Assistant) was conducted prior to the study to determine the number of experimental units per group required to compare the lung viral RNA values. An n number of 4 animals per group was calculated, with a power of 0.8 and a significance level of 0.05. The minimum effect size was determined as a 2-fold difference in lung viral RNA, with a standard deviation of 0.38 derived from previous comparable in-house studies. Statistical comparison for the percentage weight change between the sham treatment group and the treatment groups was conducted using a two-way ANOVA multiple comparison with Bonferroni correction. A nonparametric Mann–Whitney test (one-tailed) was conducted to statistically determine the differences in the viral RNA load (nasal turbinate and lungs) between the sham-treated and the treatment groups, as well as the differences in the viral RNA load (nasal turbinate and lungs) and SARS-CoV-2 viral titres (lungs) between the treatment groups (FVP vs. RDV, FVP vs. FVP + RDV, and RDV vs. FVP + RDV). An unpaired t-test (two-tailed) was used to compare the percentage area of viral antigen in the lung section between the sham treatment group and the treatment groups. Significance was determined by *p* ≤ 0.05 for all statistical comparisons (GraphPad Prism, v 10.0.2).

## 3. Results

### 3.1. Weight Changes in Each Treatment Group Across the Study Period

**Figure 1:**
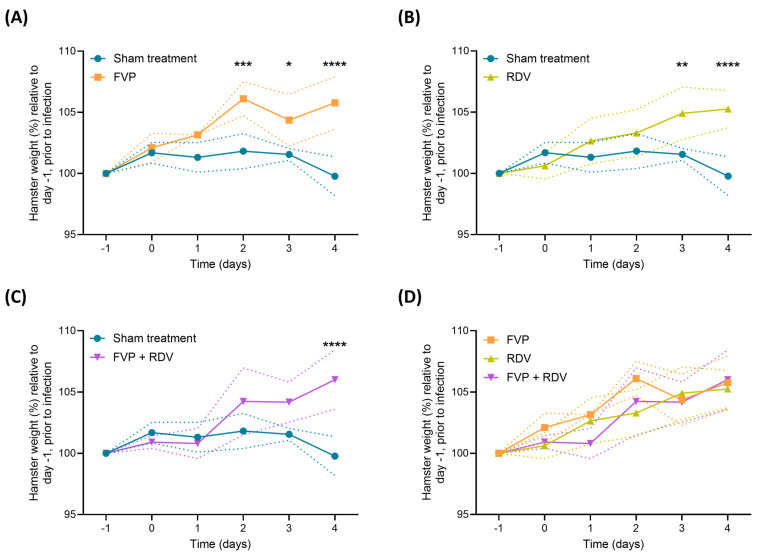
Percentage weight change of each treatment group throughout the entire study duration. Hamsters in each treatment group (n = 4) were weighed every day from day −1 to day 4. Weights are represented as a percentage of the initial weight measured at the beginning of the study, on day −1. (**A**) Sham treatment vs FVP, (**B**) sham treatment vs RDV, (**C**) sham treatment vs FVP + RDV, and (**D**) FVP vs. RDV vs. FVP + RDV. Two-way ANOVA multiple comparison with Bonferroni correction was used to determine statistical significance. * = *p* ≤ 0.05, ** = *p* ≤ 0.01, *** = *p* ≤ 0.001, **** = *p* ≤ 0.0001. Dotted lines represent standard deviations.

All animals were weighed daily throughout the study to follow the clinical course of the disease. In Figure 1, the average weight at each time point for every group is represented as a percentage in relation to the average weight on day −1 before infection within that specific group. Animals in the sham treatment group showed a weight increase at day 0 (101.7%), which stayed constant until day 3 (101.6%), and then declined by 1.8% at day 4 (99.8%). Across the study, weights increased in the FVP, RDV, and FVP + RDV treatment groups compared to their respective weights at day −1 (for all these groups, average weight gain of 5.7% by day 4). The group receiving the FVP treatment showed an increase in weight starting from day 0, with their weights significantly higher than those of the sham treatment group from day 2 onwards (day 2: *p* = 0.0002; day 3: *p* = 0.0212; day 4: *p* ≤ 0.0001; Figure 1A). The group treated with RDV (Figure 1B) exhibited a consistent body weight increase starting from day 1, with weights significantly higher than those of the sham treatment group by day 3 (*p* = 0.0078) and day 4 (*p* ≤ 0.0001). In the FVP + RDV treatment group (Figure 1C), the weight remained steady until day 2, and then increased up to 6% by day 4, being significantly higher compared to that in the sham treatment group (*p* ≤ 0.0001). No significant differences in weight were found between the treatment groups (FVP vs. RDV, FVP vs. FVP + RDV, and RDV vs. FVP + RDV) during the 4 days (Figure 1D).

### 3.2. Viral RNA Quantification in Nasal Turbinate and Lung Samples

**Figure 2:**
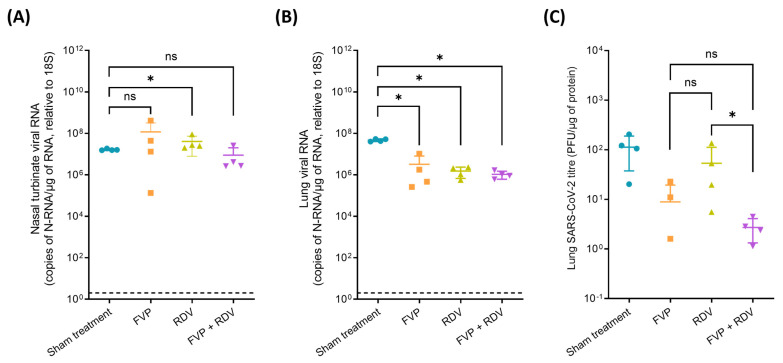
Viral quantification (N-RNA) and lung viral titres (PFU/µg of protein) of SARS-CoV-2 from samples of each treatment group (n = 4) at day 4. (**A**) Nasal turbinate and (**B**) lung copies of viral N-RNA/µg of RNA, relative to 18S, from each treatment group. Statistical significance between the sham treatment group and treated groups was determined using a nonparametric Mann–Whitney test (one-tailed, *p* ≤ 0.05). (**C**) Sham treatment group samples were processed separately from the treatment groups (hence no statistical comparison could be performed). Statistical significance between the different treatment groups (FVP vs. RDV, FVP vs. FVP + RDV, and RDV vs. FVP + RDV) was determined using a nonparametric Mann–Whitney test (one-tailed, *p* ≤ 0.05). Lung viral titres from the FVP + RDV combination group were lower in comparison to the FVP and RDV monotherapy groups but only significantly different from the latter. ns = not statistically significant, * = *p* ≤ 0.05. LOD: limit of detection (indicated by dotted line).

Viral N-RNA levels in nasal turbinate and lung samples taken at 4 dpi are shown in Figure 2. In the nasal turbinate tissue, viral N-RNA was detected in all animals (Figure 2A). On average, SARS-CoV-2 RNA values from the sham treatment group were lower than those from the FVP and RDV treated groups, being significantly different for the latter (1.6 × 10^7^ vs. 1.2 × 10^8^ copies of N-RNA/µg of RNA relative to 18S, *p* = 0.5; and 1.6 × 10^7^ vs. 4.1 × 10^7^ copies of N-RNA/µg of RNA relative to 18S, *p* = 0.014, respectively). The average SARS-CoV-2 RNA value from animals treated with the FVP + RDV combination therapy was lower compared to the value obtained from the sham treatment group but not significantly different (1.6 × 10^7^ vs. 8.9 × 10^6^ copies of N-RNA/µg of RNA relative to 18S, *p* = 0.17). Furthermore, the viral N-RNA levels in nasal turbinate in the FVP + RDV combination-treated group were not statistically different to those in either the FVP (*p* = 0.24) or the RDV (*p* = 0.057) treated groups.

In the lungs, SARS-CoV-2 RNA was detectable in all animals in all groups. As shown in Figure 2B, viral RNA levels in the sham treatment group were significantly higher than those in FVP (4.7 × 10^7^ vs. 3.2 × 10^6^ copies of N-RNA/µg of RNA relative to 18S, *p* = 0.014), RDV (4.7 × 10^7^ vs. 1.5 × 10^6^ copies of N-RNA/µg of RNA relative to 18S, *p* = 0.014), and FVP + RDV (4.7 × 10^7^ vs. 1.1 × 10^6^ copies of N-RNA/µg of RNA relative to 18S, *p* = 0.014) treatment groups. Pulmonary viral RNA in the FVP + RDV combination group did not differ significantly different from that in either the FVP (*p* = 0.5) or the RDV (*p* = 0.24) group.

Supplementary Table S1 contains data on the copies of N-RNA/µg of RNA relative to 18S in individual animals on day 4. Additionally, Supplementary Tables S2 and S3 present details on the average copies of N-RNA/µg from all treatment groups and the p-values from their comparison.

### 3.3. Live Virus Quantification via Plaque Assay in Lung Samples Obtained at Day 4 Post Infection

As shown in Figure 2C, SARS-CoV-2 viral titres were on average higher in the sham treatment group (113 PFU/µg of protein) than in the FVP (9 PFU/µg of protein), RDV (54 PFU/µg of protein), and FVP + RDV combination (3 PFU/µg of protein) groups. Furthermore, in the case of the FVP group, no viral titres were detected in 1 of the 4 hamsters (#1). However, since the samples from the sham treatment group were processed separately from the treatment groups, a statistical comparison could not be performed. SARS-CoV-2 titres did not differ significantly between the FVP group and the FVP + RDV combination group (9 vs. 3 PFU/µg of protein, *p* = 0.4429, Table S3); however, the titres in the FVP + RDV combination group were significantly lower than in the RDV group (54 vs. 3 PFU/µg of protein, *p* = 0.0143, Table S3).

### 3.4. Histological Evaluation and Viral Antigen Expression

The histological and immunohistological examination of the lungs confirmed widespread infection in sham-treated hamsters at day 4 post infection (Figure S2A). Infection was associated with focal parenchymal alterations, represented by activation of type II pneumocytes and alveolar macrophages, the presence of some degenerate alveolar epithelial cells, and some syncytial cells or focal desquamation of alveolar epithelial cells and alveolar macrophages, as well as leukocyte infiltration, dominated by macrophages and lymphocytes, with some neutrophils. Mild vasculitis and perivascular leukocyte infiltration was also observed (Figure S3A). Viral antigen expression was seen in respiratory epithelial cells (variable numbers of intact and occasionally degenerate epithelial cells in bronchioles) and type I and II pneumocytes, both within unaltered and altered parenchymal areas, and occasional macrophages in the latter (Figure S2A). Animals in the three treatment groups exhibited a similar pattern of viral antigen expression, though with less widespread infection (Figure S2B–D). The histological changes were generally minimal and represented by a mild increase in interstitial cellularity and patchy type II pneumocyte activation (Figure S3B–D). Detailed information on the results in individual animals is provided in Supplemental Table S1.

**Figure 3:**
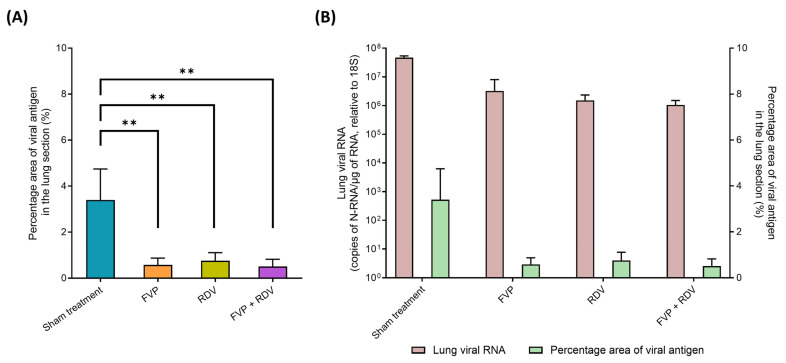
Morphometric analysis to compare the extent of viral antigen expression in the different treatment groups. (**A**) The extent of viral antigen expression is determined as percentage area of viral nucleocapsid protein (NP) expression in the area covered by the lung section. Statistical significance between the sham treatment group (n = 4) and the corresponding treated group (n = 4) was determined using an unpaired t-test (two-tailed, *p* ≤ 0.05). ** = *p* ≤ 0.01. (**B**) Correlation of viral N-RNA levels (as copies of N-RNA/µg of RNA, relative to 18S) and the extent of viral NP expression (as percentage area of viral antigen in the lung section).

A morphometric analysis served to quantify and compare the extent of SARS-CoV-2 NP expression in the left lung (the right lung had served for viral RNA quantification and plaque assay) as a further means to compare the extent of viral infection in the different groups. As shown in Figure 3A, the percentage area of viral antigen immunolabelling in relation to the total area in the lung sections was significantly higher (3.40% on average) in the sham treatment group than in the FVP group (0.58%; *p* = 0.0004), the RDV group (0.76%; *p* = 0.0007), and the FVP + RDV combination group (0.51%; *p* = 0.0003) but did not differ significantly between the monotherapy and combination groups (Table S4). As seen in Figure 3B, the SARS-CoV-2 N-RNA levels and the extent of viral antigen expression in the lung were positively correlated.

## 4. Discussion

Both FVP and RDV have been repurposed for the treatment of SARS-CoV-2 infection with varying quality of clinical evidence [6,7]. FVP and RDV in combination might improve efficacy, and the current study sought to evaluate FVP, RDV, and FVP + RDV administered intraperitoneally to Syrian golden hamsters infected with SARS-CoV-2 (B.1.1.7).

In rodents, weight changes reflect the clinical course of SARS-CoV-2 infection [27]. The hamsters in the sham treatment group maintained the same weight for the first 3 days after infection and had begun to lose weight when they were culled at day 4 post infection. This differs to what is observed in untreated mock infected hamsters, where animals gain weight daily [28] but is in line with what has previously been reported in hamsters infected with SARS-CoV-2 at a low inoculum [15]. As was expected, at day 4 the viral infection was confirmed in all hamsters from the sham treatment group by all the methods used in the study. Animals from this group were positive for viral RNA in the nasal turbinates and lungs, and exhibited SARS-CoV-2 viral titres and widespread infection in the lungs, as shown by immunohistology. Moreover, our results confirm that infection with the B.1.1.7 variant leads to histological changes and viral infection similar to those seen with the ancestral G614 strain, inducing a comparable disease and immune response [17].

The FVP group showed weight gain after intranasal infection with SARS-CoV-2, with weights consistently significantly higher than those in the sham treatment group from 2 days post infection onwards. All animals from the FVP group were positive for viral RNA in the nasal turbinate and lung. While the viral RNA was higher compared to the sham treatment group in the nasal turbinate, the viral RNA in lung tissue was significantly lower. This tendency in the lung was also observed in the plaque assay, histological examination, and morphometric analysis. Indeed, the PFU/µg of protein value was 13 times lower in the FVP group compared to the sham treatment group, and infectious virus was undetectable in one of the animals (#1 FVP). In line with this, infection of the lung in the FVP group was less widespread and associated with significantly less intense viral antigen expression, as shown by immunohistology and morphometry, with minimal histological changes. Previous experimental reports have shown that the same (300 mg/kg/day, orally) or higher (1400 mg/kg/day, intraperitonially) dose of FVP may reduce infectious virus titres in lungs, but no weight gain was evidenced throughout these studies [15,29], even showing significant weight loss from the first day of treatment [29]. Whether the early weight loss was due to a toxic effect of the drug was not determined. However, in the current study, the histological examination did not reveal any evidence of pulmonary adverse effects after treatment with FVP.

In general, RDV had a comparable effect to that of FVP. Body weights in the RDV group increased consistently from day 1 and were significantly higher than in the sham treatment group at days 3 and 4. The viral RNA in the nasal turbinate and lung was significantly higher and lower, respectively, in comparison to that in the sham treatment group. Plaque assay results also showed lower viral titres in the RDV-treated group in comparison to the sham treatment group (4.1-fold). Similar findings have been previously reported using the same dose of RDV and route of administration (15 mg/kg, intraperitoneal) [30]. In these previous studies, hamsters were therapeutically dosed from day 1 to 3 with RDV after being intranasally infected with 10^5^ PFU of SARS-CoV-2 Lineage A at day 0. Despite a viral challenge 10^3^ higher than the current study, the viral titres were lower both in nasal wash and the lung in comparison to the sham-treated group, but only significantly different in the lung at day 4 [30]. Less widespread infection and a significant difference in the extent of SARS-CoV-2 NP expression in the left lung between RDV and sham treatment groups aligned well with the results obtained by PCR and plaque assay in the right lung. Most of the results showed a beneficial effect of RDV in comparison to the sham treatment group, except for the viral RNA measured in the nasal turbinates. This unexpected result might be explained by the small sample size (n = 4 per group). A larger sample size, especially in the sham treatment group, may provide more robust statistical power [31].

In the FVP + RDV combination group, while weights had started to increase by day 2, they were only significantly higher than in the sham treatment group at day 4, at which point this group exhibited the highest body weight gain of all groups (6% weight gained since day −1). This combination group was the only group in which all outcome measures (viral RNA in nasal turbinates and right lung, viral titres in the right lung, and the extent of viral antigen expression in the left lung) were lower than in the sham treatment group. Indeed, the values obtained for the FVP + RDV combination group were the lowest of all study groups (Tables S2–S4). Nonetheless, like for the monotherapies, the differences were only significant for the lungs. Like the current report, previous studies have observed that FVP (orally) and RDV (subcutaneously) mainly reduced infection and replication in lung tissue, but not at the nasal level [15]. Interestingly, intranasal administration has been previously observed to reduce infection in nasal the turbinate and lung for FVP, RDV, pibrentasvir, and nafamostat [12,13].

An overwhelming statistical benefit of the FVP + RDV combination was not observed over FVP or RDV monotherapies in this study but is challenging to detect when both single agents provide virological efficacy. While viral RNA, viral titres, and the extent of viral antigen expression were lower after FVP + RDV combination treatment, the difference was only significant for the viral titres, as determined by plaque assay, and only in comparison to the RDV group. Similar challenges have been noted by other investigators who have assessed drug combinations. A previous study also evaluated the combination of FVP (300 mg/kg, orally) and GS-441524 (metabolite of RDV, 25 mg/kg, subcutaneously) in hamsters infected with 10^1^ or 10^3^ PFU of SARS-CoV-2 Lineage A [15]. The results showed a significant reduction in the virus titres in the lungs and nasal turbinate compared to the control and GS-441524 monotherapy groups, but not in comparison to the FVP monotherapy group. Another report determined the combined efficacy of suboptimal doses of molnupiravir and FVP [32]. Hamsters were treated BID with molnupiravir (150 mg/kg, orally) + FVP (300 mg/kg, intraperitoneally) from day 0 (one hour before infection with 2 × 10^6^ TCID50 of SARS-CoV-2 BetaCov/Belgium/GHB-03021/2020 (EPI ISL 109 407976|2020-02-03) to 3 dpi. The viral RNA levels, viral titres, and pathology scores observed in the lungs for the combination group were all significantly lower compared to the FVP monotherapy group, but compared to the molnupiravir monotherapy group, significant differences were only achieved in the virus titres. Despite the challenge of discriminating efficacy, there are other benefits that are expected to accrue for drug combinations. Firstly, the use of combinations dramatically reduces the potential for emergence of drug resistance because of the need to simultaneously acquire different resistance mutations for all of the drugs in the regimen. Secondly, a combination regimen may be more robust in the face of emerging variants of a virus, which may be more compromised for one drug in the regimen than for others. Finally, different drugs penetrate different tissue and cellular compartments to different degrees, and one may anticipate better antiviral coverage across compartments for a combination compared to a single agent even though this is extremely difficult to assess empirically.

One limitation of the present study is that the drug therapies were dosed before inoculation with virus, and thus the outcomes are not reflective of treatment but rather may be more applicable to chemoprophylaxis. Since remdesivir is administered clinically as an intravenous preparation, it is not a medicine that is well suited to prophylaxis and is best suited to administration in the hospital setting. However, several groups have developed different prodrugs of analogues of the remdesivir active metabolite, to develop orally administrable medicines [33,34]. Thus, future studies should assess the effectiveness of FVP + RDV in treatment (including with immunocompromised animals), and/or assess the combination of FVP with novel oral analogues based upon remdesivir active metabolites. Another limitation to consider is that the efficacy of the combination was assayed only against the SARS-CoV-2 Alpha variant. Both drugs have maintained relatively constant in vitro activity against different variants, which have predominantly differed with respect to the sequence of the spike protein and not the polymerase. However, subsequent studies should consider sequence changes in the polymerase should they occur in future variants.

In the current report, we did not evaluate a sham infection/sham treatment group and sham infection/drug treatment groups because previous studies that have used similar doses and/or the same route of administration as those applied in the current study, both for FVP [15,29], and RDV [14,15,35,36,37], did not report any toxicological effect related to the use of these drugs in hamsters. Specifically, it has been demonstrated that an intraperitoneal injection of 75 mg/day of FVP in non-infected hamsters (a dose 2.5-fold higher than the one used in the current manuscript, 30 mg/day) leads to evidence of toxicity; this was not observed with a dose between 18.75 and 37.5 mg/day [29]. In the case of RDV, no signs of toxicity have been reported using the same dose used in the present manuscript (1.5 mg/day, intraperitoneal) [14,35,36], or even higher (2.5 mg/day, subcutaneous) [15,37].

Combination therapies should improve efficacy through synergistic and/or additive effects [11], with the benefit of delaying the selection of viral drug resistance [38,39]. Hence, while the present study did not holistically yield statistically significant evidence that FVP + RDV in combination was superior to individual agents, it seems logical to conclude that both nucleosides are incorporated by the SARS-CoV-2 polymerase without detriment.

## Data Availability

The data presented in this study are available upon request from the corresponding author or can be found in Supplementary Figures S1–S3 or in Tables S1–S4.

## Supplementary Materials

The following supporting information can be downloaded at: https://www.mdpi.com/article/10.3390/v16121838/s1, Figure S1: Study design for evaluation of the efficacy of favipiravir (FVP), remdesivir (RDV), or FVP + RDV to block SARS-CoV-2 infection. The drugs were administered intraperitoneally. RDV or FVP was dosed once a day (OD) or twice a day (BID), respectively. The sham treatment group was dosed intramuscularly with sucrose and HPMC only at day −1. Treatments started 24 h prior to intranasal infection with 1 × 10^2^ PFU SARS-CoV-2 B.1.1.7 Alpha and continued until day 3. All animals were culled at day 4; Table S1: Relevant histological changes and SARS-CoV-2 nucleoprotein expression in Syrian hamsters after intranasal infection with 10^2^ PFU SARS-CoV-2 B.1.1.7 Alpha and euthanised at 4 days post infection; Table S2: Average viral RNA levels (copies of N-RNA/µg of RNA relative to 18S) in the nasal turbinate (NT) and right lung (Lung) at day 4 post infection, for sham treatment and groups treated with FVP, RDV, and FVP + RDV. The p-value of each group in comparison to the sham treatment group (Sham vs. Treatment), FVP group (FVP. vs. Treatment), and RDV group (RDV. vs. Treatment) is shown in the table. Significantly different comparisons are shown in bold (* = *p* ≤ 0.05, nonparametric Mann–Whitney test, one-tailed). An arrow indicates if the difference between one treated group and another treatment (i.e., Con. vs. Treatment) is significantly higher (↑) or lower (↓); Table S3: Average SARS-CoV-2 viral titre (PFU/µg of protein) in the right lung at day 4 post infection for sham treatment and groups treated with FVP, RDV, and FVP + RDV. The p-value of each group in comparison to the FVP group (FVP. vs. Treatment), and RDV group (RDV. vs. Treatment) is shown in the table. Significantly different comparisons are shown in bold (* = *p* ≤ 0.05, nonparametric Mann–Whitney test, one-tailed). ND: not determined. An arrow indicates if the difference between one treated group and another treatment (i.e., vs. Treatment) is significantly higher (↑) or lower (↓); Figure S2: Viral antigen expression in the lung of hamsters at day 4 post intranasal infection with 10^2^ PFU of SARS-CoV-2 (lineage B.1.1.7 Alpha variant); Figure S3: Histopathological features in the lung of hamsters at day 4 post intranasal infection with 10^2^ PFU of SARS-CoV-2 (lineage B.1.1.7 Alpha variant); Table S4: Average percentage area of viral nucleocapsid protein (NP) expression in the area covered by the lung section at day 4 post infection for sham treatment and groups treated with FVP, RDV, and FVP + RDV. The p-value of each group in comparison to the FVP group (FVP. vs. Treatment), and RDV group (RDV. vs. Treatment) is shown in the table. Significantly different comparisons are shown in bold (* = *p* ≤ 0.05, unpaired parametric t-test, two-tailed). An arrow indicates if the difference between one treated group and another treatment (i.e., Con. vs. Treatment) is significantly higher (↑) or lower (↓).
