# Supplementary material for: Assessment of Favipiravir and Remdesivir in Combination for SARS-CoV-2 Infection in Syrian Golden Hamsters
Source: Viruses. 2024 Nov 27;16(12):1838. doi: 10.3390/v16121838 (PMC11680105; doi:10.3390/v16121838)
Supplement: Supplementary file 1 [file viruses-16-01838-s001.zip › viruses-3309402-supplementary.pdf]

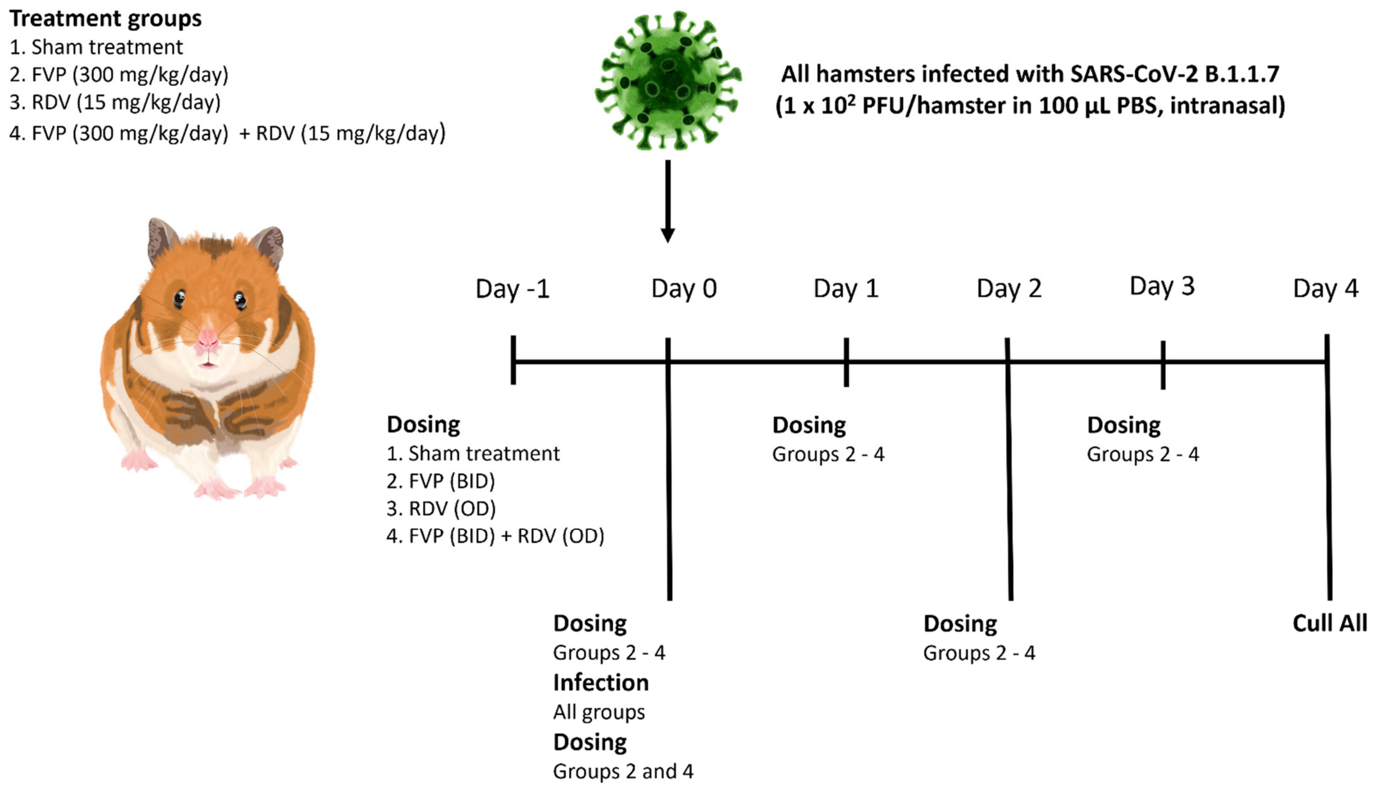


**Figure S1.** Study design for evaluation of the efficacy of favipiravir (FVP), remdesivir (RDV), or FVP + RDV to block SARS-CoV-2 infection. The drugs were administered intraperitoneally. RDV or FVP was dosed once a day (OD) or twice a day (BID), respectively. The sham treatment group was dosed intramuscularly with sucrose and HPMC only at day -1. Treatments started 24 hours prior to intranasal infection with $1 \times 10^2$ PFU SARS-CoV-2 B.1.1.7 Alpha and continued until day 3. All animals were culled at day 4.

**Supplementary Table S1.** Relevant histological changes and SARS-CoV-2 nucleoprotein expression in Syrian hamsters after intranasal infection with $10^2$ PFU SARS-CoV-2 B.1.1.7 Alpha and euthanised at 4 days post infection.

| **Animal No**[1] | **Treatment** | **Histological changes and viral antigen expression (left lung)** | **Virology**[2] **(PCR; plaque assay)** |
|---|---|---|---|
| 1.1 | Sham | **Lungs**: large focal consolidated area with type II pc and AM activation and infiltrating leukocytes (macrophages, LC, some NL), some deg and syncytial cells, a few further smaller patches; mild vasculitis and pv leukocyte infiltration<br>**vAg**: widespread patches of alveoli with pos AEC (-50% of parenchyma); individual to large patches of pos BEC in bronchus and larger bronchioles | N: 15426285<br>L: 51947545;<br>106 |
| 1.2 | Sham | **Lung (HE)s**: several large, partly coalescing areas with type II pc and AM activation and infiltrating leukocytes (macrophages, LC, some NL), some deg and syncytial cells, a few further smaller patches; mild vasculitis and pv leukocyte infiltration<br>**vAg**: MF to coalescing patches of alveoli with pos AEC; individual to large patches of pos intact and occ deg BEC in larger bronchioles | N: 15908677<br>L: 39943983;<br>205 |
| 1.3 | Sham | **Lung (HE)**: several areas with type II pc and AM activation and infiltrating leukocytes (macrophages, LC, some NL), some deg and syncytial cells, a few further smaller patches; mild vasculitis and pv leukocyte infiltration<br>**vAg**: several, partly larger patches of alveoli with pos AEC; individual up to large patches of pos intact and occ deg BEC in larger bronchioles | N: 15615606<br>L: 53771834;<br>122 |
| 1.4 | Sham | **Lung (HE)**: several areas with type II pc and AM activation and infiltrating leukocytes (macrophages, LC, some NL), some deg and syncytial cells, focal looser areas with AEC desquamation and some syncytial cells; mild vasculitis and pv leukocyte infiltration<br>**vAg**: multiple, mainly small patches of alveoli with pos AEC; individual to large patches of pos intact and occ deg BEC in larger bronchioles | N: 18762697<br>L: 42422283;<br>20 |
| | | | |
| 2.1 | Favipiravir | **Lungs**: mild increase in interstitial cellularity; patchy type II pc activation<br>**vAg**: bronchioles with a few to large patches of intact and occ deg pos BEC; with mainly adjacent, variably sized patches of alveoli with pos AEC | N: 413011680<br>L: 464541;<br>negative |
| 2.2 | Favipiravir | **Lungs**: mild mf increase in interstitial cellularity;<br>**vAg**: bronchioles with a few to large patches of intact and occ deg pos BEC; a few random, rather small patches of alveoli with pos AEC | N: 133289<br>L: 259306;<br>2 |
| 2.3 | Favipiravir | **Lungs**: mild mf increase in interstitial cellularity; patchy type II pc activation<br>**vAg**: bronchioles with a few to numerous intact and occ deg pos BEC; several random, mod sized patches of alveoli with pos AEC | N: 43950021<br>L: 10349426;<br>23 |
| 2.4 | Favipiravir | **Lungs**: mild mf increase in interstitial cellularity; patchy type II pc activation<br>**vAg**: a few individual to small groups of pos ep cells in some bronchioles; several random, mod. sized patches of alveoli with pos AEC | N: 13049003<br>L: 1773086;<br>11 |
| | | | |
| 3.1 | Remdesivir | **Lungs**: mild mf increase in interstitial cellularity; patchy type II pc activation<br>**vAg**: a few individual to small groups of pos EC in bronchus and a few bronchioles; a few random small patches of alveoli with pos AEC | N: 27574731<br>L: 564303;<br>20 |
| 3.2 | Remdesivir | **Lungs**: mild mf increase in interstitial cellularity; patchy type II pc activation<br>**vAg**: bronchus with rare individual pos intact EC, a few bronchioles with individual to small groups of pos EC; several variably sized patches of alveoli with pos AEC | N: 24962679<br>L: 2319522;<br>136 |
| 3.3 | Remdesivir | **Lungs**: mild mf increase in interstitial cellularity; rel. widespread type II pc activation<br>**vAg**: bronchioles with a few to numerous intact and occ deg pos BEC; with mainly adjacent, variably sized alveoli with pos AEC | N: 89345654<br>L: 2094634;<br>53 |
| 3.4 | Remdesivir | **Lungs**: mild mf increase in interstitial cellularity; widespread type II pc activation<br>**vAg**: a few bronchioles with a few to a patch of intact and occ deg pos BEC; with a few mainly adjacent, mainly large patches of alveoli with pos AEC | N: 20269276<br>L: 1051138;<br>6 |
| | | | |

| | | | |
|---|---|---|---|
| 4.1 | Favipiravir + Remdesivir | **Lungs**: mild mf increase in interstitial cellularity; patchy type II pc activation<br>**vAg**: bronchus with numerous pos intact and occ deg EC, also a few bronchiole with several pos EC and others with viral antigen lining ep cells; one adjacent, mod sized patch of alveoli with pos AEC | N: 4117060<br>L: 909076;<br>1 |
| 4.2 | Favipiravir + Remdesivir | **Lungs**: mild mf increase in interstitial cellularity; patchy type II pc activation<br>**vAg**: bronchus and a few bronchiole with several pos intact and occ deg EC; a few mod sized, mainly adjacent patches of alveoli with pos AEC | N: 25959486<br>L: 608980;<br>2 |
| 4.3 | Favipiravir + Remdesivir | **Lungs**: mild mf increase in interstitial cellularity; patchy type II pc activation<br>**vAg**: bronchus and a few bronchiole with several to numerous pos intact and occ deg EC; a few small to mod sized, mainly adjacent patches of alveoli with pos AEC | N: 2738671<br>L: 1040528;<br>5 |
| 4.4 | Favipiravir + Remdesivir | **Lungs**: mild mf increase in interstitial cellularity; patchy type II pc activation<br>**vAg**: bronchus and several bronchioles with often numerous pos intact and occ deg EC; several small to mod sized, mainly adjacent patches of alveoli with pos AEC | N: 2632151<br>L: 1664422;<br>3 |

**Legend**: AEC – alveolar epithelial cells; AM – alveolar macrophages; AT – airborne transmission; BEC – bronchiolar epithelial cells; deg – degenerate; d – day; EC – epithelial cells; HE – histological features assessed in a hematoxylin-eosin stained section; IP – intraperitoneal; LC – lymphocyte; mf – multifocal; mode – moderate; neg – negative; NHA – no histological abnormality; NL – neutrophils; NT – nasal turbinates; LC – lymphocytes; pb – peribronchiolar; pc – pneumocytes; pos – positive; pv – perivascular; vAg – viral antigen

[1] Treatment groups: Group 1 –intramuscular injection of vehicle (150 µL) into each thigh at day -1. Group 2 – intraperitoneal injection of 150 mg/kg of favipiravir twice daily, from day -1 through to day 4. Group 3 – intraperitoneal injection of 15 mg/kg remdesivir once daily, from day -1 through to day 4. Group 4 – intraperitoneal injection of 150 mg/kg of favipiravir twice daily and 15 mg/kg remdesivir once daily, from day -1 through to day 4.

[2] Virology: PCR (copies of viral N-RNA/µg of RNA relative to 18S) determined in nasal turbinates (N) and right lung (L); plaque assay (SARS-CoV-2 viral titre in PFU/µg of protein) determined in tissue from the right lung

**Supplemental Table S2.** Average viral RNA levels (copies of N-RNA/µg of RNA relative to 18S) in the nasal turbinate (NT) and right lung (Lung) at day 4 post infection, for sham treatment and groups treated with FVP, RDV, and FVP + RDV. The p value of each group in comparison to the sham treatment group (Sham vs. Treatment), FVP group (FVP. vs. Treatment), and RDV group (RDV. vs. Treatment) is shown in the table. Significantly different comparisons are shown in bold (* = P ≤ 0.05, nonparametric Mann-Whitney test, one-tailed). An arrow indicates if the difference between one treated group and another treatment (i.e. Sham vs. Treatment) is significantly higher (↑) or lower (↓).

| Group | Tissue | Average viral RNA | Sham vs. Treatment | FVP vs. Treatment | RDV vs. Treatment |
|---|---|---|---|---|---|
| Sham | NT | $1.6 \times 10^7$ | X | P = 0.5 | ***P = 0.014 ↑** |
| | Lung | $4.7 \times 10^7$ | X | ***P = 0.014 ↓** | ***P = 0.014 ↓** |
| FVP | NT | $1.2 \times 10^8$ | P = 0.5 | X | P = 0.44 |
| | Lung | $3.2 \times 10^6$ | ***P = 0.014 ↑** | X | P = 0.34 |
| RDV | NT | $4.1 \times 10^7$ | ***P = 0.014 ↓** | P = 0.44 | X |
| | Lung | $1.5 \times 10^6$ | ***P = 0.014 ↑** | P = 0.34 | X |
| FVP + RDV | NT | $8.9 \times 10^6$ | P = 0.17 | P = 0.24 | P = 0.05 |
| | Lung | $1.1 \times 10^6$ | ***P = 0.014 ↑** | P = 0.5 | P = 0.24 |

**Supplemental Table S3.** Average SARS-CoV-2 viral titre (PFU/µg of protein) in the right lung at day 4 post infection for sham treatment and groups treated with FVP, RDV, and FVP + RDV. The p value of each group in comparison to the FVP group (FVP. vs. Treatment), and RDV group (RDV. vs. Treatment) is shown in the table. Significantly different comparisons are shown in bold (* = P ≤ 0.05, nonparametric Mann-Whitney test, one-tailed). ND: not determined. An arrow indicates if the difference between one treated group and another treatment (i.e. Sham vs. Treatment) is significantly higher (↑) or lower (↓).

| Group | Average PFU/µg of protein | Sham vs. Treatment | FVP vs. Treatment | RDV vs. Treatment |
|---|---|---|---|---|
| **Sham** | 113 | X | ND | ND |
| **FVP** | 9 | ND | X | P = 0.1 |
| **RDV** | 54 | ND | P = 0.1 | X |
| **FVP + RDV** | 3 | ND | P = 0.4429 | ***P = 0.0143 ↑** |

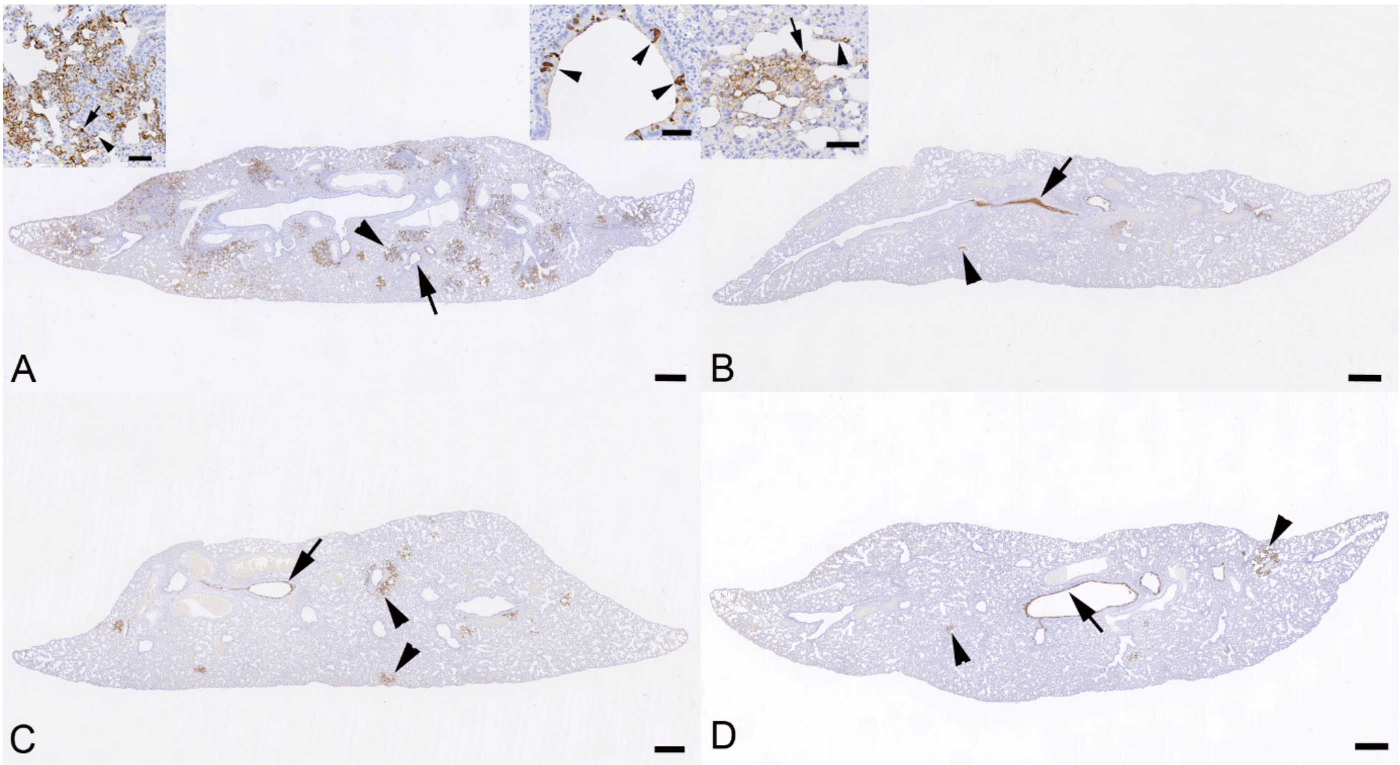


**Figure S2.** Viral antigen expression in the lung of hamsters at day 4 post intranasal infection with $10^2$ PFU of SARS-CoV-2 (lineage B.1.1.7 Alpha variant). The arrows in the overview images highlight bronchioles with viral antigen expression in epithelial cells, and the arrowheads highlight parenchymal areas with viral antigen expression in alveolar epithelial cells. (A) Sham treatment hamster group; animal 1.4. There is widespread viral antigen expression in multiple disseminated patches of alveoli. Left inset: focal area (highlighted by arrowhead in large image) with leukocyte infiltration and viral antigen expression in type I (arrowhead) and type II (arrow) pneumocytes. Right inset: bronchiole (highlighted by arrow in large image) with viral antigen expression in individual intact epithelial cells. (B) Hamster treated with FVP; animal 2.2. Viral antigen expression is restricted to a large bronchiole (arrow) and a focal patch of alveoli (arrowhead). Inset: Focal area highlighted by arrowhead in large image, showing viral antigen expression in type I (arrowhead) and type II (arrow) pneumocytes. (C) Hamster treated with RDV; animal 3.3. Viral antigen expression is seen in a bronchiole (arrow) and a few patches of alveoli (arrowheads). (D) Hamster treated with FVP + RDV; animal 4.3. Viral antigen expression is seen in a few bronchioles (arrow) and rare patches of alveoli (arrowheads). Immunohistology for SARS-CoV-2 nucleoprotein, hematoxylin counterstain. Bars = 500 µm (large images) and 50 µm (insets).

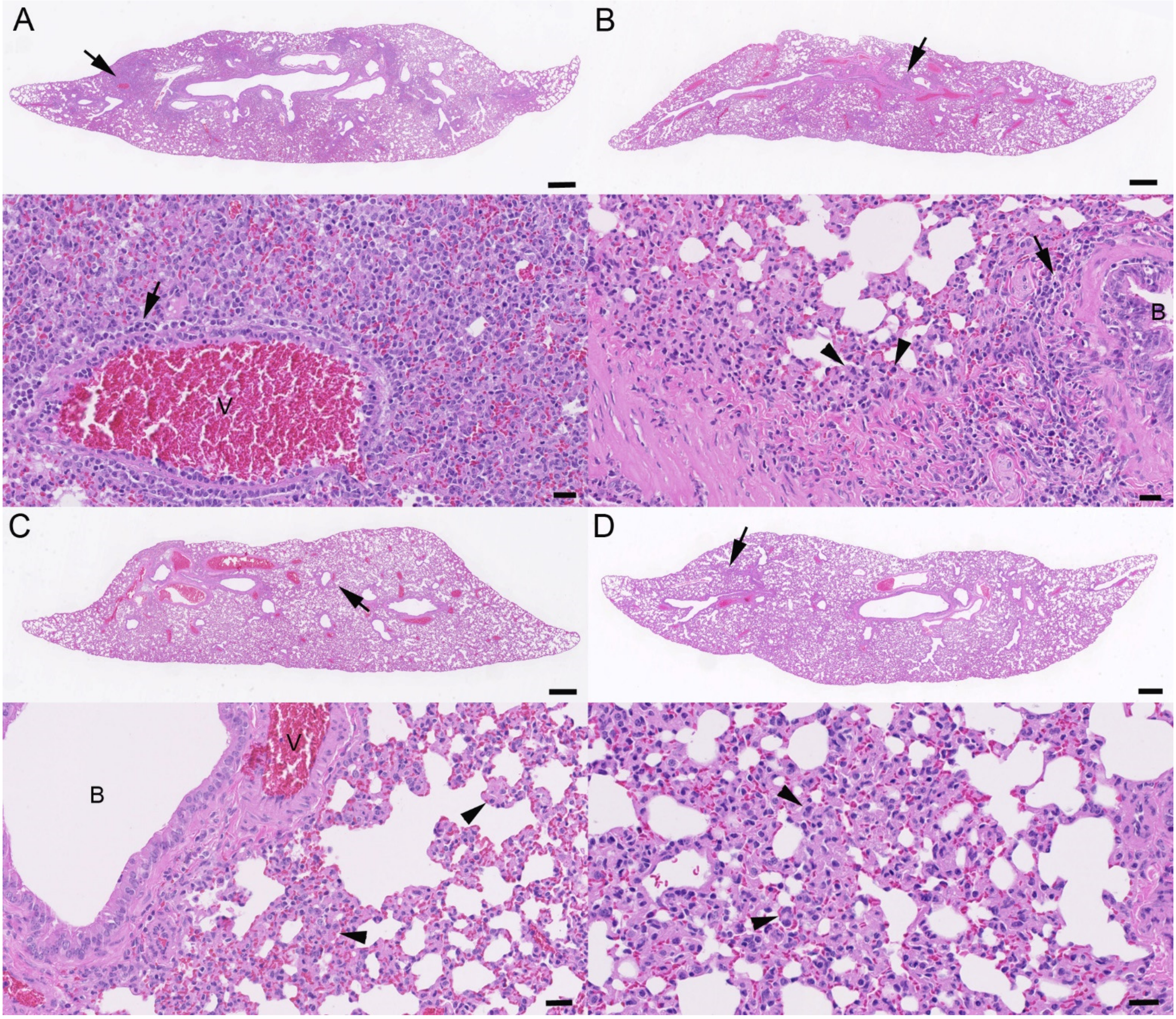


**Figure S3.** Histopathological features in the lung of hamsters at day 4 post intranasal infection with $10^2$ PFU of SARS-CoV-2 (lineage B.1.1.7 Alpha variant). The arrows in the overview images highlight the area shown in detail in the higher magnifation below. (A) Sham treatment hamster group; animal 1.4. There are multiple consolidated parenchymal areas (arrow). The higher magnification shows a consolidated area with SARS-CoV-2 infection (see Fig. S2A), with abundant activaed type II pneumocytes, alveolar macrophages and leukocyte infiltration. The arrow points at subendothelial and perivascular leukocyte infiltration of a vessel (consistent with vasculitis). (B) Hamster treated with FVP; animal 2.2. The lung parenchyma is widely unaltered, with small focal areas of increased interstitial cellularity. The higher magnification shows an area adjacent to an infected bronchiole (see Fig. S2B) of increased interstitial cellularity, with activated type II pneumcytes (arrowheads; the left arrowhead highlights a syncytial cells). There is also mild peribronchial mononuclear infiltration (arrow). (C) Hamster treated with RDV; animal 3.3. The lung parenchyma is widely unaltered, with very small focal areas of increased interstitial cellularity. The higher magnification shows an area of SARS-CoV-2 infected alveoli (see Fig. S2C) with activated type II pneumcytes (arrowheads). (D) Hamster treated with FVP + RDV; animal 4.3. The lung parenchyma is widely unaltered, with very small focal areas of increased interstitial cellularity. The higher magnification shows an area with activated type II pneumcytes (arrowheads). B: bronchiole; V: vessel. Hematoxylin eosin stains. Bars = 500 µm (overviews) and 25 µm (closer views).

**Supplemental Table S4.** Average percentage area of viral nucleocapsid protein (NP) expression in the area covered by the lung section at day 4 post infection for sham treatment and groups treated with FVP, RDV, and FVP + RDV. The p value of each group in comparison to the FVP group (FVP. vs. Treatment), and RDV group (RDV. vs. Treatment) is shown in the table. Significantly different comparisons are shown in bold (* = P ≤ 0.05, unpaired parametric t-test, two-tailed). An arrow indicates if the difference between one treated group and another treatment (i.e. Con. vs. Treatment) is significantly higher (↑) or lower (↓).

| Group | Average % Area | Con. vs. Treatment | FVP vs. Treatment | RDV vs. Treatment |
|---|---|---|---|---|
| **Sham** | 3.40 | X | ***P = 0.0064 ↓** | ***P = 0.0089 ↓** |
| **FVP** | 0.58 | ***P = 0.0064 ↑** | X | P = 0.47 |
| **RDV** | 0.76 | ***P = 0.0089 ↑** | P = 0.47 | X |
| **FVP + RDV** | 0.51 | ***P = 0.0058 ↑** | P = 0.74 | P = 0.33 |
